# Buffalo Academy of Medicine

**Published:** 1904-12

**Authors:** Franklin W. Barrows

**Affiliations:** Secretary


### SOCIETY PROCEEDINGS.

### Buffalo Academy of Medicine.

*Section on Medicine, October 11, 1904.*

Reported by FRANKLIN W. BARROWS, M. D., Secretary.

THE regular meeting of the medical section was held Tuesday evening, October 11, 1904, at the Academy rooms in the Public Library Building. The meeting was called to order at 8.45 o'clock by the chairman, Dr. Allen A. Jones. The minutes of the last meeting were read and approved.

Dr. Eli H. Long presented a paper, entitled

### ESSENTIAL MEDICATION IN CARDIAC DISEASES.

(Author's abstract.)

The writer aimed to discuss only common conditions and well-known remedies. He called attention to the two types of cardiac disease: (*a*) endocardial; inflammatory in nature, of which rheumatic disease is a type; (*b*) myocardial; degenerative in character, of which sclerotic or fatty heart is a type.

He reviewed briefly the physiologic action of the most commonly employed drugs.

As representing the first class of cases, acute endocarditis with valvular disease was discussed. The use of digitalis was considered improper during the acute inflammation, its action being contrary to the principle of rest and tending to aggravate the condition.

Aconite was mentioned as the drug which would give the same slowing effect as digitalis, and at the same time lessen force and friction of the blood current within the heart. The great majority of cases could be carried to perfect compensation without the use of cardiac stimulants. In the minority of cases, when the heart is unable even under conditions of rest and good hygiene to maintain a proper circulation, we have a disturbed balance between the arterial and venous sides of the circulation. With venous fulness there occurs diminished oxygenation, faulty katabolism and deficient elimination. The blood is more or less toxic and the heart is poorly nourished. Dilatation of the heart follows. These are emergency cases which call for stimulation, and the use of digitalis should be looked upon as emergency treatment. The writer emphasised the importance of dilatation and its bearing upon treatment, and considered that in such cases digitalis, by contracting the volume of the heart, places it at a better advantage for efficient action.

Elimination was regarded as of prime importance. Jalap being nonirritating and very prompt, was recommended in full doses, in order to secure from three to six fluid stools daily. Strychnine is not relied upon by the writer for much direct cardiac effect, being rather a potential nerve stimulant. Caffeine stimulates the rapidity of the heart action without increasing the efficiency. Atropine was regarded as a second rate cardiac stimulant. Cases of broken compensation become essentially myocardial, and require treatment approaching that for the second class of diseases.

Regarding the myocardial or degenerative class, the rule was laid down that, the greater the degree of degeneration the less we may expect from cardiac stimulants and the less they are indicated. The importance of the cardiac nutrition, dependent upon a good general nutrition of tissues, was emphasised. The typical case would be one associated with arteriosclerosis, inefficient capillary nutrition throughout the body, and irregularity of the heart dependent on the degree of degeneration. Nonmedicinal measures were regarded as more important than medicinal, particularly those that improve the circulation in the capillary area and lessen resistance in the circulation.

The use of digitalis in these cases was questioned, because of its vasoconstrictor effect. When needed, a vasodilator should be combined with it.

Atropine was advised in cases with slow pulse, which probably represents diminished reactive power of the heart.

The nitrites were spoken of for temporary use, to lessen peripheral resistance and improve capillary circulation, but it was pointed out that the vasodilation and the more rapid heart action were both due to a depressant action of the drug. The writer concluded by emphasising the belief that the object to be sought always is not stimulation of the heart, but improvement in its nutrition.

DISCUSSION.

The discussion was opened by Dr. Charles G. Stockton who said that in inflammatory conditions, especially endocarditis, drugs of the digitalis type are contraindicated, although in a few cases with early and rapid dilatation, digitalis may be good.

In acute endocarditis, besides rest and cutaneous stimulation of the capillaries by sinapisms, friction or heat, one of the most important steps is to combat the toxins. Eliminative measures are important. A. L. Loomis said/that by treating the great glands, like the liver, we could do the most for the heart. Large doses of calomel are of great service. Blood letting over the liver and wet cupping or leeching over the liver, give relief.

Digitalis improves the nutrition of the heart and lessens dilatation better than any other drug. But its use as a routine is regrettable. There comes a time in the disease when it is useless. It affects people very differently, and we can only know its use in a given case by making a trial of it.

In all heart cases we use too much medicine. We should relieve toxicity and open the "capillary area." Assistive respiratory movements are useful.

Digitalis is often valuable in spite of degenerative changes in the heart, especially when given with the vasomotor dilators. Nitroglycerine as commonly employed does very little good as a vasomotor dilator. By giving doses in strength just short of causing headache, every six hours, we get poor results. It should be given every hour or two, in minute doses—1-300 to 1-500 grain.

Dr. Henry R. Hopkins said he had often succeeded in the treatment of greatly dilated heart by the use of morphine hypodermatically. Aconite, in large doses—10 to 30 drops of the tincture—two or three times in twenty-four hours reduces blood pressure from 190 or 200 to 150 or 160.

DR. A. L. BENEDICT read a paper entitled,

### CLASSIFICATION OF GASTRIC ULCERS.

(Author's abstract.)

He urged the use of the word ulcer as applied to the stomach in the same general sense of a solution of superficial continuity, in which it is used for other parts of the body, especially as there is no unanimously accepted conception of what is ordinarily termed peptic ulcer.

The diagnosis of gastric ulcer is intimately connected with hemorrhage. Indeed, without hemorrhage or the opportunity of direct inspection, we can scarcely make more than a tentative diagnosis of gastric ulcer and, conversely, a hemorrhage that can clinically be ascribed to the stomach, almost always indicates some form of ulcer of the stomach. The following classification, therefore, not only includes a broad conception of gastric ulcer but, for convenience, includes some forms of hematemesis not strictly arising from an ulcer in any true sense of the word.

1. Peptic ulcer. This occurs mainly in neurotic and fairly young persons, mostly women. It is probably due to spasm of arterioles or an opposite condition of paralysis, as in Reynaud's disease and almost certainly not to organic vascular disease, embolism and the like, as formerly taught. The primary element in the production of any true gastric ulcer is death of tissue or so marked depression of vitality that digestion of the area affected occurs. Such digestion is an inevitable consequence, unless there is a marked achylia. While peptic ulcer is usually connected with hyperchlorhydria, it should not be assumed that there is merely an erosion by acid and, indeed, it is by no means established that hyperchlorhydria and peptic ulcer are typically associated.

2. Superficial erosions due to chemic and thermic corrosion.

3. Ulcers due to organic vascular lesions, such as embolism, thrombosis. The frequency of end arteries in the stomach favors the production of necrotic areas, whether the obstruction to circulation be neurotic or organic.

4. Catarrhal ulcers are, without propriety, separated from the last group, but the vascular disease is less definite and local. They occur in chronic gastritis, on account of diminished nutrition of the gastric cells and are analogous to certain eczematous ulcers of the skin.

5. Varicose ulcers are usually due to portal obstruction and, practically, almost always to portal obstruction or hepatic obstruction. There may be massive, even fatal hemorrhage, without time for the development of a true ulcer and the hemorrhage may occur from ruptured veins of the esophagus or intestine, so that the exact diagnosis and classification may be difficult. Packard, shortly before his death, collected about sixty cases. The writer had seen about a dozen, only one of which was included in Packard's list, so that the condition is more common than might appear. When the hemorrhages are small and repeated, it may be difficult to diagnose this condition from cancerous ulcer. On the other hand, persons with ulcers of the third and fourth type may also have hepatic sclerosis, so that the differential diagnosis is often impossible during life.

6. Toxemic diapedesis, with a tendency to actual rhexis, occurs in scurvy, purpuras, and perhaps in Banti's disease. This is not a true ulcer, although it is possible that one may develop secondarily.

7. Vicarious menstruation is commonly recognised as a cause of gastric hemorrhage. It is not a true ulcerative condition according to the ordinary conception but the pathology of the condition is subject to considerable argument.

8. Gangrenous ulceration of the stomach occurs when, for some reason, there is a failure of prompt proteolytic digestion. The ultimate cause of such ulceration might, theoretically, be very varied and, indeed, gangrenous ulceration is not really to be considered as a separate group. The writer had never seen a case except in cancer of the stomach, so that he considered a putrid odor coming from the empty stomach or other evidence of actual bacterial decomposition of necrotic tissue in masses, as fairly diagnostic of cancer, though, of course, not pathognomonic.

9. Phlegmonous ulceration is essentially due to the action of bacteria, either beginning as an ulcer with burrowing of pus or beginning with some form of abscess which ruptures into the stomach, producing an ulcer. When not easily traced to pyemia or to some extragastric septic focus, phlegmonous ulceration of the stomach is most commonly due to iodine poisoning.

10. Specific ulcers of the stomach include those due to various exanthematous, granulomatous and neoplastic processes.

11. Traumatic ulcers may be due to external violence, penetrating or crushing, or to internal lesions by hard particles, foreign bodies, or gross parasites. The wounds thus inflicted may or may not persist so as to become true ulcers.

In conclusion, Dr. Benedict showed a stomach with a large kidney-shaped ulcer on the greater curvature, due to arteriosclerosis. The existence of the ulcer had been determined by hemorrhage about four months before death. The immediate cause of death was shock, due to perforation through the mesocolon and into the greater peritoneal cavity, the contents of the peritoneum having shown peptonising power postmortem. Another specimen was shown in which the clinical diagnosis of hour glass stomach had been made, by auscultatory percussion and *x*-ray shadow. The specimen verified this diagnosis so far as contour was concerned but a more careful, histologic examination would be necessary to determine whether there was a genuine hour glass division of the stomach or whether the constriction, through which the opening barely admitted a slate pencil, was at the pylorus, the lower cavity in that case being the dilated duodenum.

DISCUSSION.

The discussion was opened by Dr. HENRY R. HOPKINS, who said that the question is of the greatest obscurity. Accurate notions of gastric ulcer are difficult to attain, and in the majority of cases are reached only after autopsy. A few of the questions at present unsolved are: (1) is gastric ulcer increasing in frequency? (2) is it directly related to hyperacidity due to hydrochloric acid? (3) can it be accurately diagnosticated? (4) is there an acute form? (5) is it rare in the upper classes of society? The doctor thought that pathologic micro-organisms play a very large part in the causation of this disease, because certain infections,—tuberculosis, syphilis, endocarditis,—are likely to be associated with gastric ulcer. Dr. Hopkins referred to a case of a woman who died from gastric hemorrhage after confinement, probably caused by the strain of the labor. There is no disease in which the expectant treatment is so nearly a crime as in gastric ulcer.

Dr. STOCKTON would not agree with Dr. Benedict as to the importance of hemorrhage. We cannot make a positive diagnosis without hemorrhage, neither can we make a diagnosis because of hematemesis.

Dr. DOWD related the case of a woman in whom a mistaken diagnosis of gastric ulcer was made. She was hysterical.

Dr. ALLEN A. JONES emphasised the trophic nature of gastric ulcer. A part of the stomach may be so injured by trophic conditions as to allow peptic action to attack it.

In closing the discussion, Dr. BENEDICT said that he realised that in suggesting a different use of terms to that in vogue, he had invited criticisms, but that clinical experience was forcing the necessity of recognising the existence of other forms of solution of continuity of the gastric wall, than that commonly termed peptic ulcer. Certainly, it could not be true that the very class of persons in whom cerebral apoplexy and embolism, calcification, arteriosclerosis and similar lesions in general were rare, should have organic vascular disease of the stomach. On the other hand, ulcers of the stomach are common, both ante- and postmortem, in which there is nothing to suggest the ordinary peptic ulcer. As to the bacterial cause of gastric ulcer, he had not undertaken to enter into the ultimate causation of ulcers in general, except so far as was necessary to the classification. In many cases, possibly in all, the toxemia due to bacteria might be the underlying cause of the loss of vitality of the cells, but this point had not been demonstrated and he was not prepared to accept it as a general theory. So, too, bacteria might participate in the destruction of necrotic areas, and they certainly did in the case of gangrenous and phlegmonous ulcerations. But, as to the pathogenesis of gastric ulcer in general and, especially, of the ordinary peptic ulcer, there was no question but that the actual excavation of the ulcer was due to peptic digestion. As to the connection between ulcer and hyperchlorhydria, the condition contraindicated the use of the tube for exact diagnostic purposes and the theory that hyperchlorhydria produces peptic ulcer is not supported by a sufficient number of quantitative examinations. In many instances, such conclusions have been based on very crude methods. On the contrary, we do know that ulcer sometimes develops in patients who have previously shown diminished hydrochloric acidity, and that hyperchlorhydrics usually fail to develop an ulcer.

Attendance at the meeting, 44. Adjournment at 10.35 p. m.

---

### *Section on Medicine, November 8, 1904.*

REPORTED BY FRANKLIN W. BARROWS, M. D., Secretary.

THE regular meeting of the medical section was held in the Academy rooms, Public Library Building, Tuesday evening, November 8, 1904. In the absence of the chairman the meeting was called to order at 8.50 o'clock by the president of the Academy, DR. ARTHUR W. HURD. The minutes of the previous meeting were read and approved.

The paper of the evening was presented by DR. W. GILMAN THOMPSON, professor of medicine in the Cornell University Medical College, New York City, entitled,

#### PROBLEMS IN DIETETICS.

(Abstract.)

The paper discussed certain problems, such as the feeding of patients in whom a combination of diseases might appear to demand diametrically opposite systems of diet; the evils which may result from too long continued restriction in diet, such as weakness and anemia; the diet of obscure nutritional disorders such, for example, as arthritis deformans; and the prophylactic diet of such diseases as gout and rheumatism.

The author advocated the early feeding of typhoid fever patients with semisolid food, in suitable cases—namely, very mild cases in general; those in which a low grade of fever persists for many days in spite of improvement in the condition of the tongue, the stools, the abdomen, and the mental symptoms; cases in which a rapid loss of weight with low temperature appears to be the most serious symptom, and in uncomplicated cases on the first day of normal temperature. Relapses, he said, are rarely, if ever, attributable to carefully regulated increase in the diet, but, on the contrary, such increase may better enable the patient to withstand a relapse.

In arthritis deformans the diet should resemble that of the early stages of phthisis, consisting largely of fats and animal foods, and effort should be directed toward maintenance of nutrition by every means, including forced feeding.

In chronic nephritis, especially when complicated by cardiac disease, too long continuance of a milk diet is liable to increase the anemia and weaken the heart muscle. It is better, therefore, in many cases to allow a moderate quantity of meat in the diet, with eggs, fish and other lighter forms of animal food. The patient's weight, strength and degree of anemia constitute safer guides for feeding than the urinalysis alone. When chronic nephritis is complicated by a diabetes which demands an opposite dietetic system, it should be determined which disease appears to threaten life the most, and then establish the proper regimen for that disease. For example, in a man past fifty years of age, a moderate glycosuria may be disregarded in the presence of the greater danger of a serious chronic nephritis, with a pulse of high tension and diminished proteid elimination. The amount of fluid ingested in chronic nephritis with anasarca should be regulated by the pulse tension and urine elimination. With too much fluid the heart may be overtaxed and arterial tension raised; whereas, with too little, there is danger of retention of the urinary waste products, and the plugging of the renal tubules with casts. Hence, in each case the quantity should be adapted from time to time to changing conditions.

In the dietetic treatment of diabetes the writer disregards entirely the use of gluten flours and breads, as being unreliable in composition and unsatisfying to the patient, whose natural craving for starches is not appeased by the innutritious gluten. Potatoes contain, bulk for bulk, less than one half as much starch as wheaten bread, and may often be allowed to the extent of a couple of ounces a day; or one or two small slices of bread may be eaten twice a day. In any well developed case periods of total abstinence from starches and sugars should be made to alternate with their moderate allowance, as improvement in the urine and general symptoms ensues. If the patient continues to lose weight and forms sugar out of his own body proteids, large quantities of fat foods should be eaten, such as butter, cream, bone marrow, lard and suet used in cooking, olive oil, fat meats and fat fish, such as mackerel or salmon, and sardines soaked in oil.

Too rigid adherence to the so-called "antidiabetic diet" is often productive of more harm than good through starvation and anemia, and a better rule is to allow the moderate use of bread and potatoes as long as the patient appears to be in "nitrogenous equilibrium," that is, not consuming his own tissues and emaciating, and in all cases fat foods should be ingested in considerable quantity.

Lithemia demands a temporary vegetable and fruit diet, with large quantities of water, until proteid waste is thoroughly eliminated. Return to a diet of animal food should be made very gradually after ten days or a fortnight. Patients should be cautioned against overtaxing their nerve force through excessive exercise, and exercise should be definitely regulated, together with periods of rest, both before and after meals.

The influence of diet upon arteriosclerosis was discussed and, although it is admitted that mental and physical strain appear to be the chief etiological factors in many nontoxic cases, it also appears desirable in such cases to restrict the overeating of proteid food, and to regulate fluid ingestion in relation to arterial tension and the work of the heart.

In the treatment of goutiness between acute attacks of gout, of greater importance than the abandonment of occasional articles of food in a long dietetic list, are the following general principles: first, to reduce the consumption of food as a whole; second, to increase the consumption of water; third, to eliminate entirely sugars and sweets of every kind, as well as alcohol; and fourth, to reduce the consumption of red meats to a minimum.

The *ad interim* or prophylactic dietetic treatment of acute rheumatism is distinctly disappointing, and if this disease is an infection, as there are good grounds for believing, there is little reason for seeking aid in dieting, except in the milk diet of the acute attacks. A diet designed to prevent anemia and maintain a high standard of nutrition is the best preventive, and to this end animal food must be largely included.

Although the paper purposely omitted discussion of the dietetic treatment of diseases of the alimentary canal, the writer protested against the common fallacy of drawing conclusions for dietetic rules from a single gastric analysis; for repeated analyses in the same case often show all variations from anacidity to hyperchlorhydria. The common test meal of a roll and glass of water is not sufficiently appetising to always excite gastric secretion; or, the latter may be inhibited by the dread of the passage of the stomach tube.

DISCUSSION.

Dr. Charles G. Stockton, in opening the discussion, said he had never heard so much common sense applied to a scientific subject as in this paper. Dr. Thompson's views express the results of scientific observation as well as clinical study. He was unable to endorse the views of the paper on the early feeding of solids in typhoid. His own experiences warn him to wait until the temperature has been normal for some time. As to nephritis, he agrees with the paper. The best diet in this case is that which is best for the liver. As is the primary digestion, so is the liver; as is the liver, so, usually, is the kidney. The paper is right in pointing out the unwisdom of using gluten breads in diabetes. If the patient can assimilate fats they are useful; but to a considerable class of diabetics the fats are toxic. If oxybutyric and diacetic acids are abundant in the urine it is best to drop fats from the dietary. The speaker agreed with the ideas of the paper on lithemia.

Dr. Frederick C. Busch said that there is a lack of experimental research in the metabolism of various diseased conditions. Such work, along the lines of some existing physiologic studies in metabolism, would be useful, but difficult to accomplish. The speaker had observed cases of typhoid fever in which purpuric or scorbutic symptoms appeared under a milk diet. Peptones and broths improved the condition. Recent researches, in Harvard University, with *x*-rays, indicate that when food passes the ileocecal valve there is an antiperistaltic wave; later on, the bowel contents are carried onward by peristalsis. Food introduced by rectum may be carried into the small intestine by the antiperistaltic wave; hence rectal feeding is unlikely to protect typhoid ulcers.

Dr. DeLancey Rochester, in reply to the last speaker, said that he never resorted to rectal feeding until the stomach refused to retain food. In typhoid he had often found the yolk of egg useful. Predigested milk with orange juice added is seldom refused by the patient. The speaker had made many gastric analyses in cases of arthritis deformans, and found that in dieting this disease we must rely on the patient's ability to digest. In nephritis, when the fluid is not absorbed from the tissues and cavities, salt starvation is a useful treatment, as well as deprivation of fluids. Professor Chittenden's recent researches in nutrition, at Yale University, throw great light on the treatment of lithemia, showing that the chief point in treatment is the reduction of the total amount of food. Hard work is the chief factor in causation of arteriosclerosis; another important factor is the lack of excretion. The laboring classes, especially, neglect the skin; this predisposes to gout also. In treating mucous enteritis and enterocolitis we must recognise that the nervous system is chiefly at fault. We must exclude irritating foods from the diet and study the stools carefully.

Dr. Irving P. Lyon, referring to the statement in the paper that white meats are more easily digested than red meats, quoted the published researches of von Noorden, Frankfort-on-Main, which go to prove that the latter contain no higher percentage of extractives than the former.

Dr. Thompson, in closing the discussion, said that he had given his personal experience in the feeding of typhoid cases. In laying down rules for students to follow he would prefer to err on the side of safety and prescribe a milk diet until well into convalescence. The significance of oxybutyric and diacetic acids is a new subject, not sufficiently understood. If they are increased by fats we must reduce the fats in the diet. But we must be guided by the bedside experiences also. In a recent fatal case of diabetic coma at the Presbyterian Hospital, New York, there was no oxybutyric or acetic acid present. Green vegetables were purposely not alluded to in the paper. They are chiefly useful by adding variety to the diet, by preventing scurvy and like diseases by virtue of their organic acids and iron, and by combating constipation. Referring to Dr. Busch's remarks he said that he also had studied the mechanism of peristalsis experimentally, and he thought the results of *x*-ray study of this subject were not of clinical value. Clinically, we know that we can keep up life by rectal absorption. In reply to Dr. Lyon, the speaker said he was familiar with von Noorden's work. Our question, however, is not a laboratory question, but a question of what the patient can get out of certain kinds of food; bedside observations are more useful than laboratory analyses in such cases. As to the question of water drinking, suggested by Dr. Lyon, it is difficult to make rules. Most people drink too little water; hence the benefit of visiting spas.

Dr. Rochester moved a vote of thanks to Dr. Thompson for his exceedingly interesting and instructive paper. Carried.

The two other papers announced for this meeting were omitted by courtesy of the authors, who preferred to give place to Dr. Thompson.

Total attendance, 63. Meeting adjourned at 10.30 p. m.

